# Enhancement of the antifungal activity of some antimycotics by farnesol and reduction of *Candida albicans* pathogenicity in a quail model experiment

**DOI:** 10.14202/vetworld.2022.848-854

**Published:** 2022-04-08

**Authors:** Nadezhda Sachivkina, Alexander Senyagin, Irina Podoprigora, Elena Vasilieva, Olga Kuznetsova, Arfenia Karamyan, Alfia Ibragimova, Natalia Zhabo, Maria Molchanova

**Affiliations:** 1 Department of Microbiology and Virology, Institute of Medicine, Peoples’ Friendship University of Russia (RUDN University), 117198 Moscow, Russia; 2 Department of Biochemistry, Institute of Medicine, Peoples’ Friendship University of Russia (RUDN University), 117198 Moscow, Russia; 3 Department of Veterinary Medicine, Agrarian Technological Institute, Peoples’ Friendship University of Russia (RUDN University), 117198 Moscow, Russia; 4 Department of General Pharmaceutical and Biomedical Technologies, Institute of Medicine, Peoples’ Friendship University of Russia (RUDN University), 117198 Moscow, Russia; 5 Department of Foreign Languages, Institute of Medicine, Peoples’ Friendship University of Russia (RUDN University), 117198 Moscow, Russia

**Keywords:** amphotericin B, antimycotics, *Candida albicans*, clotrimazole, farnesol, fluconazole, intraconazole, ketoconazole, miconazole, nystatin, quail model, quorum sensing, voriconazole

## Abstract

**Background and Aim:**

Clinical strains of microorganisms, including pathogenic yeast-like fungi (YLF), are resistant to currently used antifungal agents. Thus, it is relevant to study the combinations of existing antimicrobial drugs and a medicinal extract of plant origin (farnesol). In previous studies, farnesol showed a relatively strong anti-biofilm effect against *Candida albicans*. This study aimed to determine how much the resistance profile of non-biofilm microorganisms can change.

**Materials and Methods:**

Six clinical isolates of *C. albicans* and one reference strain were used to study the interaction of farnesol with the most used antimycotics. To determine the sensitivity of YLF to antimycotic drugs, such as nystatin (50 μg), amphotericin B (10 μg), ketoconazole (10 μg), clotrimazole (10 μg), voriconazole (10 μg), fluconazole (25 μg), miconazole (10 μg), and intraconazole (10 μg), the classic disk diffusion method was used. In the second stage, one of the six strains was used to simulate candidiasis of the gastrointestinal tract in an *in vivo* quail model. As an unusual experimental design, this study investigated the effects of pretreated *C. albicans* in quails, not the *in vivo* pathogenicity of *C. albicans*, after treatment with farnesol.

**Results:**

The resistance profiles of *Candida* strains did not improve with farnesol in all strains. All concentrations of farnesol (100, 50, and 25 μM) demonstrated a fungistatic effect (i.e., an increase in drug sensitivity) in 23 of 56 (7×8) cases (41%). The remaining 54% demonstrated no changes in the resistance to antifungal drugs or deterioration of the indicators in rare cases (5%). At 100 μM farnesol, sensitivity improved in 33 of 56 cases (59%). Candidiasis or the severity of clinical disease of the quail digestive tract developed to a lesser extent if fungi were treated with farnesol.

**Conclusion:**

Farnesol does not always show a positive result on single cells without biofilm in the laboratory. However, in a biofilm or an *in vivo* model with biofilms, farnesol can be considered a new antimycotic drug or an additive to existing antimycotics.

## Introduction

*Candida albicans* is a conditionally pathogenic yeast-like fungus (YLF) that threatens a human’s weakened immune system [1,2]. Because isolated strains are resistant to currently used antifungal agents, developing new antimicrobial drugs is necessary. In the previous studies, farnesol showed a relatively strong antibiofilm effect against *C. albicans*, and the main mechanism was probably associated with the suppression of hyphal development [3-5].

In biological communities, microorganisms use various mechanisms for their communication. Depending on the cell density, bacteria and YLF can produce quorum sensing (QS) signaling molecules (e.g., secondary metabolites) involved in regulating gene expression and coordinating collective behavior in their natural niche. These secondary metabolites play a major role in the adhesion and colonization of host tissues and surfaces, morphogenesis, and biofilm development [6,7]. In yeast and YLF, farnesol plays a major role in the morphological transition, inhibiting hyphal production depending on the concentration. Tyrosol performs the opposite function, stimulating the transition from spherical to germ tube-shaped cells [8,9].

Farnesol is continuously produced in biofilms at temperatures from 23°C to 43°C and in quantities approximately proportional to colony-forming units per milliliter. Chemically, farnesol is acyclic sesquiterpene alcohol endogenously synthesized by the ergosterol pathway. Its synthesis depends on neither the type of carbon or nitrogen source nor the chemical nature of the nutrient medium, and it is a thermally stable molecule not exposed to extreme pH values [10].

Farnesol is obtained in natural and synthetic ways. Farnesol can be isolated from linden-colored essential oils, musk grains, neroli, and pettigrain. In yeast, farnesol is a by-product of the ergosterol biosynthesis pathway formed due to the enzymatic dephosphorylation of farnesyl pyrophosphate. The enzymes of this pathway are encoded by ergosterol genes. In bacteria, phytoene/squalene synthase (YisP) acts as a phosphatase, catalyzing the formation of farnesyl diphosphate. Wang *et al*. [11] described that the accumulation of farnesyl diphosphate could be used for the industrial production of farnesol in *Escherichia coli*. They found that phosphatidate phosphatase (PgpB) and undecaprenyl diphosphatase (YbjG), two integral membrane phosphatases of *E. coli*, can hydrolyze farnesyl diphosphate into farnesol [12]. Large-scale farnesol production can also be achieved through chemical synthesis and metabolic engineering in prokaryotes.

The question is why study the QS farnesol molecule? The list of antifungal agents is huge, and new drugs appear every year. However, the sensitivity of microorganisms to new drugs is weak, variable, and unequal. In addition, tests for sensitivity to antimycotics, unfortunately, are not considered a routine procedure, not always available, and usually not considered a standard technique in patient management. As practice shows, resistance studies are prescribed by doctors only in cases of deep mycoses, mainly caused by non-albicans *Candida* species. In these cases, studying whether farnesol affects changes in the sensitivity of clinical strains of *Candida* to antifungal drugs is especially interesting.

This study aimed to prove that farnesol can increase the antifungal activity of some antimycotics and can reduce *C. albicans* pathogenicity in quail model experiments.

## Materials and Methods

### Ethical approval

The study was conducted according to the guidelines of the Declaration of Helsinki and approved by the Peoples’ Friendship University of Russia (RUDN University) ethical committee (EC1/351, 05/06/2021).

### Study period and location

The study was conducted from May to July 2021. The samples were processed at the Department of Microbiology and Virology, Medical Institute, Peoples’ Friendship University of Russia, Moscow, Russia**.**

### Strains

Clinical strains of six *C. albicans* isolates (C1-C6) were isolated from women with vulvovaginal candidiasis. The identification of microorganisms was carried out using the matrix-activated laser desorption/ionization technology “Bruker Daltonik MALDI Biotyper” (Bruker Daltonik, Inc., Germany). A score of >2000 was considered reliable. The reference strain (C7) was *C. albicans* ATCC 10231. Colonies of diurnal cultures of *C. albicans* from Saburo agar (Difco, France) were washed off with physiological solution (PhS; pH 7.0). The YLF concentration was 0.5 according to McFarland, corresponding to 1.5×10^8^ cells/mL [13-16].

### Reagents

Farnesol (trans-farnesol) was purchased from Sigma-Aldrich (Germany): Molar mass 222.37 g/mol, mass of the substance 0.886 g/mL, Ns=The amount of the substance in moles = 0.886:222.37=0.004 M/mL or 4,000 μM/mL. Three concentrations of farnesol (100, 50, and 25 μM) were used, and PhS (pH 7.0) was used as the control.

### Changing the sensitivity to antimycotics with farnesol

To determine the sensitivity of YLF to antimycotic drugs (HiMedia, India), nystatin (NS; 50 μg), amphotericin B (AP; 10 μg), ketoconazole (KT; 10 μg), clotrimazole (CC; 10 μg), voriconazole (VOR; 10 μg), fluconazole (FU; 25 μg), miconazole (MIC; 10 μg), and intraconazole (IT; 10 μg), the classic disk diffusion method was used.

(a) 25 μL PhS+farnesol were applied to sterile paper disks, corresponding to a farnesol concentration of 100 μM; (b) 25 μL PhS was applied to sterile paper disks; (c) 25 μL PhS was applied to the disk with the antimycotic; (d) 25 μL farnesol (100 μM) was applied to the disk with an antimycotic; (e) 25 μL farnesol (50 μM) was applied to the disk with an antimycotic; and (f) 25 μL farnesol (25 μM) was applied to the disk with an antimycotic.

The diameter of the growth suppression zone of the culture to the antimycotic was measured in accordance with the NCCLS M-44 protocol.

### Infection of quails with *C. albicans* ATCC 10231

#### Animals

Female Texas white broiler quails (albino white pharaoh or Texas white giant), 21 days old, 30 heads, body weight 350-370 g, were used. Birds passed veterinary control and had all documentation. Quails were quarantined for 7 days before the experiment under the supervision of a veterinarian. All animal experiments were performed in accordance with the Guide for the Care and Use of Laboratory Animals [17]. Quails were placed in a cage with five birds each, received food and water *ad libitum* and were manipulated in accordance with the Local Ethics Committee for Animal Experimentation, Peoples’ Friendship University of Russia, Moscow, Russia (protocol no. 351, June 6, 2021).

To identify the effects of farnesol on the ability of *C. albicans* to cause infection, the daily culture of YLF was washed 3 times with PhS, and the YLF concentration was 0.5 according to McFarland. Then, 25 μL of 100 μM farnesol was added to 1 mL microbial suspension (experiment), and 25 μL PhS (control) was added to another test tube. The tubes were incubated for 1 h at 37°C under constant shaking. After interaction with farnesol, YLF was washed three times with PhS [18,19]. The birds were divided into experimental and control groups (15 in each). The experimental group was infected with *C. albicans*+farnesol, and the control group was infected with *C. albicans* without farnesol.

*C. albicans* (1 mL) was given to quails through a digestive probe once daily for 5 days. The birds were killed every 5 days for five heads. The experiment lasted 20 days: 5 days for infection and 15 days for the course of the experiment. All animals injected with *C. albicans* were examined histopathologically [20].

### Statistical analysis

Statistical Package for the Social Sciences 20.0 (IBM Corp., Armonk, NY, USA) was used for statistical analyses, and a significance level of 5% was adopted. Experiments with antimycotic disks were run with three replicates per condition. Data in the table on sensitivity to antimycotics are given without ± for better table perception, and ± did not exceed 0.4.

## Results

### Changing the sensitivity to antimycotics with farnesol

In the mycological study, the strains were highly resistant to antifungal drugs *in vitro*. Especially, the clinical strain C1 showed resistance to most drugs. In the other six strains, the resistance levels differed among the groups of drugs and varied within the groups. The synergistic effect of farnesol with polyene antimycotics was as follows: farnesol (100 and 50 μM) increased sensitivity to NS in six strains, and farnesol 25 (μM) increased sensitivity to NS in four strains; farnesol (100 μM) increased sensitivity to AP (50 μM) in six strains, farnesol (50 μM) increased sensitivity to AP in four strains; and farnesol (100 μM) increased sensitivity to AP (25 μM) in six strains (Tables-1-8).

**Table 1:** Nystatin sensitivity determination of 7 *Candida albicans* strains and the effect of farnesol three concentrations on these results.

	NS					

	PhS+Far	PhS	PhS+NS	NS+100 Far	NS+50 Far	NS+25 Far
C1						
24 h	0	0	0	8	8	8
48 h	0	0	0	0	0	0
C2						
24 h	8	0	16	20	20	18
48 h	10	0	18	12	20	18
C3						
24 h	8	0	20	20	20	20
48 h	0	0	20	18	18	20
C4						
24 h	10	0	18	20	20	20
48 h	10	0	16	18	18	14
C5						
24 h	12	0	20	22	22	21
48 h	10	0	20	22	22	20
C6						
24 h	10	0	22	24	24	22
48 h	0	0	22	22	20	20
C7						
24 h	0	0	22	24	24	20
48 h	0	0	22	18	20	20

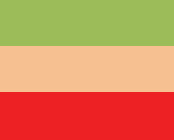
= Farnesol increases the inhibition zone = Farnesol does not affect the inhibition zone = Farnesol reduces the inhibition zone

**Table 2:** Amphotericin-B sensitivity determination of 7 *Candida albicans* strains and the effect of farnesol three concentrations on these results.

	AP					

	PhS+Far	PhS	PhS+AP	AP+100 Far	AP+50 Far	AP+25 Far
C1						
24 h	0	0	0	10	10	0
48 h	0	0	0	0	0	0
C2						
24 h	10	0	10	12	12	10
48 h	8	0	8	12	12	10
C3						
24 h	12	0	12	12	12	12
48 h	8	0	11	12	12	12
C4						
24 h	12	0	18	20	18	18
48 h	10	0	20	18	18	18
C5						
24 h	20	0	20	24	22	22
48 h	10	0	17	18	22	22
C6						
24 h	12	0	20	24	24	26
48 h	10	0	20	20	20	22
C7						
24 h	12	0	18	20	20	20
48 h	10	0	18	18	20	18

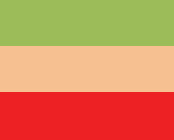
= Farnesol increases the inhibition zone = Farnesol does not affect the inhibition zone = Farnesol reduces the inhibition zone

**Table 3:** Ketoconazole sensitivity determination of 7 *Candida albicans* strains and the effect of farnesol three concentrations on these results.

	KT					

	PhS+Far	PhS	PhS+KT	KT+100 Far	KT+50 Far	KT+25 Far
C1						
24 h	0	0	0	10	12	0
48 h	0	0	0	10	10	0
C2						
24 h	14	0	10	10	10	8
48 h	12	0	8	8	8	8
C3						
24 h	12	0	12	12	12	12
48 h	9	0	9	9	6	0
C4						
24 h	8	0	12	8	8	8
48 h	0	0	10	8	8	8
C5						
24 h	10	0	22	22	22	22
48 h	10	0	16	16	18	18
C6						
24 h	12	0	12	14	18	18
48 h	12	0	12	12	18	12
C7						
24 h	12	0	18	18	16	16
48 h	12	0	14	18	12	12

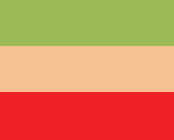
= Farnesol increases the inhibition zone = Farnesol does not affect the inhibition zone = Farnesol reduces the inhibition zone

**Table 4:** Clotrimazole sensitivity determination of 7 Candida albicans strains and the effect of farnesol three concentrations on these results.

	CC					

	PhS+Far	PhS	PhS+CC	CC+100 Far	CC+50 Far	CC+25 Far
C1						
24 h	0	0	0	0	0	0
48 h	0	0	0	0	0	0
C2						
24 h	10	0	14	16	14	14
48 h	10	0	10	12	12	14
C3						
24 h	0	0	16	16	16	16
48 h	0	0	16	16	16	16
C4						
24 h	14	0	8	14	12	12
48 h	0	0	12	10	12	12
C5						
24 h	8	0	20	20	20	20
48 h	8	0	18	18	18	20
C6						
24 h	18	0	14	18	18	18
48 h	14	0	12	14	12	16
C7						
24 h	10	0	8	16	16	14
48 h	10	0	10	16	16	14

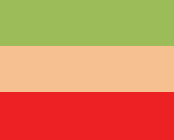
= Farnesol increases the inhibition zone = Farnesol does not affect the inhibition zone = Farnesol reduces the inhibition zone

**Table 5:** Voriconazole sensitivity determination of 7 *Candida albicans* strains and the effect of farnesol three concentrations on these results.

	VOR					
	PhS+Far	PhS	PhS+VOR	VOR+100 Far	VOR+50 Far	VOR+25 Far
C1						
24 h	0	0	0	0	0	0
48 h	0	0	0	0	0	0
C2						
24 h	10	0	18	18	20	18
48 h	0	0	8	8	8	8
C3						
24 h	14	0	20	24	22	16
48 h	10	0	20	22	22	16
C4						
24 h	14	0	16	10	0	0
48 h	10	0	14	10	10	10
C5						
24 h	12	0	16	22	28	18
48 h	10	0	14	22	26	16
C6						
24 h	14	0	16	20	20	14
48 h	12	0	12	16	18	14
C7						
24 h	12	0	12	18	14	16
48 h	12	0	0	18	14	12

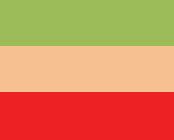
= Farnesol increases the inhibition zone = Farnesol does not affect the inhibition zone = Farnesol reduces the inhibition zone

**Table 6:** Fluconazole sensitivity determination of 7 *Candida albicans* strains and the effect of farnesol three concentrations on these results.

	FU					

	PhS+Far	PhS	PhS+FU	FU+100 Far	FU+50 Far	FU+25 Far
C1						
24 h	10	0	0	10	10	0
48 h	10	0	0	8	6	0
C2						
24 h	10	0	12	12	12	12
48 h	0	0	0	0	0	0
C3						
24 h	8	0	22	22	30	22
48 h	0	0	0	0	0	0
C4						
24 h	10	0	20	22	34	22
48 h	0	0	0	14	0	0
C5						
24 h	20	0	22	22	24	18
48 h	8	0	10	16	16	12
C6						
24 h	12	0	40+	40+	40+	40+
48 h	0	0	20	22	40+	30
C7						
24 h	15	0	40+	40+	40+	40+
48 h	14	0	0	14	16	10

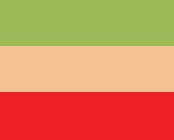
= Farnesol increases the inhibition zone = Farnesol does not affect the inhibition zone = Farnesol reduces the inhibition zone

**Table 7:** Miconazole sensitivity determination of 7 *Candida albicans* strains and the effect of farnesol three concentrations on these results.

	MIC					

	PhS+Far	PhS	PhS+MIC	MIC+100 Far	MIC+50 Far	MIC+25 Far
C1						
24 h	12	0	8	12	10	12
48 h	12	0	8	10	10	12
C2						
24 h	10	0	22	26	22	16
48 h	0	0	0	0	0	0
C3						
24 h	12	0	40+	40+	40+	40+
48 h	0	0	8	8	8	8
C4						
24 h	12	0	40+	40+	40+	40+
48 h	0	0	0	0	0	0
C5						
24 h	18	0	20	24	22	22
48 h	16	0	20	22	20	20
C6						
24 h	12	0	40+	40+	40+	40+
48 h	0	0	32	28	32	30
C7						
24 h	15	0	36	40+	40+	40+
48 h	8	0	30	30	38	26

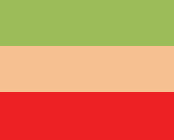
= Farnesol increases the inhibition zone = Farnesol does not affect the inhibition zone = Farnesol reduces the inhibition zone

**Table 8:** Intraconazole sensitivity determination of 7 *Candida albicans* strains and the effect of farnesol three concentrations on these results.

	IT					

	PhS+Far	PhS	PhS+IT	IT+100 Far	IT+50 Far	IT+25 Far
C1						
24 h	0	0	40+	40+	40+	40+
48 h	10	0	40+	40+	40+	40+
C2						
24 h	0	0	16	22	14	14
48 h	0	0	16	14	12	10
C3						
24 h	0	0	22	24	22	22
48 h	0	0	20	0	22	20
C4						
24 h	10	0	24	28	40+	26
48 h	0	0	24	28	40+	20
C5						
24 h	0	0	14	16	16	16
48 h	0	0	14	12	14	8
C6						
24 h	12	0	40+	40+	40+	40+
48 h	10	0	40+	40+	40+	40+
C7						
24 h	15	0	38	40+	40+	40+
48 h	10	0	28	30	40	30

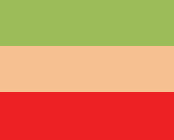
= Farnesol increases the inhibition zone = Farnesol does not affect the inhibition zone = Farnesol reduces the inhibition zone

The variability observed in the imidazole group was noteworthy. Sensitivity increased with farnesol at all three concentrations only in two strains resistant to KT. In contrast, the indicators worsened in two strains, and no changes were observed in three of seven strains. In three resistant and moderately resistant strains to CC, the resistance profiles to farnesol improved, but the resistance remained unchanged in four strains. Ambiguous results were obtained for VOR: in two cultures, farnesol increased sensitivity to antimycotic at all three concentrations; in two more strains, only the first two concentrations of farnesol (100 and 50 μM) were effective. The resistance of one clinical isolate was quite high, and farnesol even slightly worsened the indicators. Only in one strain of YLF, sensitivity to FU increased with farnesol at all three concentrations. Another culture increased sensitivity with farnesol (100 and 50 mM). In other cases, farnesol did not affect the sensitivity to FU. Sensitivity to MIC increased in three YLF cultures with farnesol (25, 50, and 100 mM). The growth retardation zone with IT in three of seven cases was higher than the control with the addition of farnesol at all three concentrations (Table-9).

**Table 9:** Summary table of mathematical calculations of the effect of farnesol to the *Candida albicans* sensitivity to antifungal drugs.

	Sensitivity improvement	Does not significantly affect sensitivity	Sensitivity deterioration
Nystatin (50 μg)	4	3	0
Amphotericin-B (10 μg)	4	3	0
Ketoconazole (10 μg)	2	3	2
Clotrimazole (10 μg)	3	4	0
Voriconazole (10 μg)	2	4	1
Fluconazole (25 μg)	2	5	0
Miconazole (10 μg)	3	4	0
Itraconazole (10 μg)	3	4	0
Total 56 (7 strains×8 antimycotics)	23 (41%)	30 (54%)	3 (5%)

### Infection of quails with *C. albicans* ATCC 10231

In the control group of birds killed within the first 5 days after infection, swelling, weak hyperemia of the goiter mucosa, thick viscous mucus and a delicate whitish plaque containing an abundance of budding cells, and pseudomycelia were observed. In the histological sections of the goiter, oral cavity, and esophagus, the start of fungal growth into the epithelial cover of the mucous membrane was detected. On the 10^th^ day after infection, curd overlays were more distinct, with rounded foci. On the 15^th^ day before the slaughter, clinical signs of the disease were observed in the form of depression, drowsiness, and poor appetite. One of the five quails remaining died. On opening, the mucous membrane of the goiter was bumpier due to the different intensities of the overlays, which in sections took the form of a solid yellow-white film, and the serous membrane became folded. Delicate, loose, and yellow-white overlays were noted on the mucous membrane of the oral cavity and tongue, in which multiple budding and pseudomycelial forms of *Candida* were detected. On the 15^th^ day, the remaining four quails killed presented with typical, well-marked candidiasis lesions in the anterior part of the digestive tract. In the histological sections, multiple filaments of the fungus were found growing perpendicular to the thickness of the mucous membrane.

In the experimental group of quails, where YLF was treated with farnesol, hyperemia of the goiter mucosa was not noticed in the first 5 days after infection. In the histological sections of the goiter, oral cavity, and esophagus, the start of fungal growth into the epithelial cover of the mucous membrane was detected but to a lesser extent than in control. On the 10^th^ day after infection on the mucous membranes of the digestive tract, a delicate whitish plaque containing a small number of budding cells was observed. On the 15^th^ day before slaughter, clinical signs of the disease in the form of depression, drowsiness, and poor appetite were not observed. Not a single quail died. On autopsy, the mucous membrane of the goiter was covered with a delicate white biofilm, in which mainly *C. albicans* budding cells were found.

This study demonstrated a significant difference in *C. albicans* whether in the form of a biofilm or individual cells not connected by a matrix. In the previous experiments, where *Candida* was in the form of a biofilm, farnesol worked perfectly. In living models, microorganisms also form biofilms. Therefore, farnesol reduces the pathogenicity of *Candida*, and the infection does not develop so rapidly and strongly. Thus, this experiment showed that *C. albicans* treated with farnesol becomes less pathogenic for animals. YLF are less able to form a biofilm under the influence of farnesol.

## Discussion

The increasing dominance of polyresistant strains of *Candida* spp. is a justification for the preliminary determination of their sensitivity to antifungal drugs and continuous mycological monitoring of the spread of these strains. Identifying such resistant strains significantly limits chemotherapy possibilities for candidiasis [1,6,21]. Treatment of candidiasis should be individual for each patient, considering the localization and severity of the process and the possibility of a chronic stage of the disease and the immune status and the presence of other diseases.

As a QS molecule, farnesol participates in regulating various physiological processes in *Candida*, including filamentation, biofilm formation, drug susceptibility, and apoptosis [3,4,16,22]. This compound is produced by many organisms, mainly fungi and is also found in many essential oils of plants [23]. The secretion of farnesol was confirmed under various conditions in eight representatives of the genus: *C. albicans*, *Candida dubliniensis*, *Candida tropicalis*, *Candida parapsilosis*, *Candida guilliermondii*, *Candida kefyr*, *Candida krusei*, and *Candida glabrata*. Whether farnesol can affect the resistance of the strain to modern drugs remains an open question. Because this process of stability or sensitivity will always be dynamic for different isolates and change over time, it will always be relevant.

Farnesol and its derivatives exhibit antibiofilm, anticancer, antitumor, and fungicidal properties [12,18,24]. The antibiofilm activity of farnesol was described depending on the administration time during the development of *Candida* biofilm and the concentration used. However, the effect of farnesol on its synergy or antagonism with known antifungal drugs has been studied to a lesser extent. This study showed that farnesol certainly affects the degree of YLF resistance, indicating its potential role as an antimicrobial agent [3,4,25,26]. However, results showed that the resistance profiles of *Candida* strains are not improved by farnesol in all strains. All concentrations of farnesol (100, 50, and 25 μM) demonstrated a fungistatic effect (i.e., an increase in drug sensitivity) in 23 of 56 (7×8) cases (41%). The remaining 54% demonstrated no changes in the resistance to antifungal drugs or deterioration of the indicators in rare cases (5%). At 100 μM farnesol, sensitivity improved in 33 of 56 cases (59%).

## Conclusion

In the previous studies [3,4], farnesol affected biofilm formation more than planktonic cells. The current study confirmed this statement in an *in vivo* model. Candidiasis of the digestive tract developed to a lesser extent if fungi were treated with farnesol. In combination with increased sensitivity to existing drugs, the effect of reducing the pathogenicity and reducing biofilm formation is a good result. We believe that the effect of farnesol on the sensitivity of *C. albicans* to antifungal drugs is successful. Our plans are to check the change in sensitivity to other drugs for this purpose. Of course, this experience will be useful for a deeper understanding of the potential antifungal mechanism of QS molecules and a continuing search for new drugs against *Candida* and other YLF.

## Data Availability Statement

The supplementary data can be available from the authors on reasonable request.

## Authors’ Contributions

AK, NS and EV: Conception and designed the study. NS, AS, and OK: Collected the samples and data analysis. IP, AI, NZ, and MM: Drafted the manuscript. All authors read and approved the final manuscript.
